# Fluid intake patterns of children and adolescents: results of six Liq.In^7^ national cross-sectional surveys

**DOI:** 10.1007/s00394-018-1725-y

**Published:** 2018-06-01

**Authors:** C. Morin, J. Gandy, R. Brazeilles, L. A. Moreno, S. A. Kavouras, H. Martinez, J. Salas-Salvadó, J. Bottin, Isabelle Guelinckx

**Affiliations:** 1 Hydration and Health Department, Danone Research, Route Départemental 128, 91767 Palaiseau, France; 2 British Dietetic Association, Birmingham, UK; 3 School of Life Medical Services, University of Hertfordshire, Hatfield, UK; 4 Biometrics and Data Science Department, Danone Research, Palaiseau, France; 5 GENUD (Growth, Exercise, Nutrition and Development) Research Group, Faculty of Health Sciences, Universidad de Zaragoza, Instituto Agroalimentario de Aragón (IA2), Instituto Investigación Sanitaria Aragón (IIS Aragón), Zaragoza, Spain; 6 CIBERobn (Centro de Investigación Biomédica en Red Fisiopatología de la Obesidad y Nutrición), Institute of Health Carlos III, Madrid, Spain; 7 Hydration Science Lab, University of Arkansas, Fayetteville, AR USA; 8 Division of Endocrinology, University of Arkansas for Medical Sciences, Little Rock, AR USA; 9 Hospital Infantil de México Federico Gómez, México City, Mexico; 10 Human Nutrition Unit, Hospital Universitari de Sant Joan de Reus, Faculty of Medicine and Health Sciences, Institut d’Investigació Sanitària Pere Virgili, Biochemistry and Biotechnology Department, Universitat Rovira i Virgili Reus, Reus, Spain

**Keywords:** Beverages, Fluid intake, Water, Hydration, Liq.In^7^, Children, Adolescents, Clustering analysis

## Abstract

**Purpose:**

This study aimed to identify and characterize patterns of fluid intake in children and adolescents from six countries: Argentina, Brazil, China, Indonesia, Mexico and Uruguay.

**Methods:**

Data on fluid intake volume and type amongst children (4–9 years; *N* = 1400) and adolescents (10–17 years; *N* = 1781) were collected using the validated 7-day fluid-specific record (Liq.In^7^ record). To identify relatively distinct clusters of subjects based on eight fluid types (water, milk and its derivatives, hot beverages, sugar-sweetened beverages (SSB), 100% fruit juices, artificial/non-nutritive sweetened beverages, alcoholic beverages, other beverages), a cluster analysis (partitioning around k-medoids algorithm) was used. Clusters were then characterized according to their socio-demographics and lifestyle indicators.

**Results:**

The six interpretable clusters identified were: *low drinkers–SSB* (*n* 523), *low drinkers–water and milk* (*n* 615), *medium mixed drinkers* (*n* 914), *high drinkers–SSB* (*n* 513), *high drinkers–water* (*n* 352) and *very high drinkers–water* (*n* 264). Country of residence was the dominant characteristic, followed by socioeconomic level, in all six patterns.

**Conclusions:**

This analysis showed that consumption of water and SSB were the primary drivers of the clusters. In addition to country, socio-demographic and lifestyle factors played a role in determining the characteristics of each cluster. This information highlights the need to target interventions in particular populations aimed at changing fluid intake behavior and improving health in children and adolescents.

**Electronic supplementary material:**

The online version of this article (10.1007/s00394-018-1725-y) contains supplementary material, which is available to authorized users.

## Introduction

Low fluid intake, or its biomarkers, has been associated with an increased risk of developing cardiometabolic diseases [1, 2], chronic kidney disease [3] and recurrent kidney stones [4]. Adequate hydration has been shown to improve cognition in children [5] and adults [6], improve mood [7, 8] and attenuate biological risk factors for the above-mentioned conditions. In addition, consuming sugar-sweetened beverages (SSB) has been linked with an increased future risk of obesity [9], and cardiometabolic diseases. Each daily serving increase in SSB has been linked to a 21% increased risk of type 2 diabetes [10], 7% increased risk of hypertension [11] and 7–18% increased risk of stroke, heart failure and coronary heart disease [12].

Total fluid intake has been reported for many countries [13–20] together with data on the type of fluid consumed [21–25]. Many studies have shown that a high proportion of children and adolescents do not drink enough to meet water intake recommendations [13, 15, 16, 23]. This is especially important in young children who, due to their relatively high body water content and underdeveloped regulatory systems, are vulnerable to the effects of not drinking enough [26]. It is apparent from these, and many other studies, that while an individual may be drinking sufficient in terms of volume to meet, or exceed current recommendations on fluid intake, there may be a wide variety of combinations of fluid type within that total volume. Considering only one or two variables at a time limits the interpretation of data, and as a consequence, their usefulness [27]. Increasingly, dietary patterns are being studied to investigate these interactions [28]. Therefore, it is now pertinent to look at patterns of fluid consumption as opposed to studies that consider only volume or individual fluid type.

Different methods of analyzing dietary patterns can be used in diverse populations, including principal component analysis, cluster analyses and more recently, reduced rank regression [29]. Fluid intake has been evaluated using multiple regression analysis [19], principal component analysis [30] and a dynamic panel model [31]. Another multivariate technique that can be applied to fluid intake is cluster analysis. The main advantage of cluster analysis is that it creates groups of individuals that are as homogeneous as possible, minimizing the variance within each group and maximizing the variance between groups. This allows the identification of fluid intake patterns, called ‘clusters’, common to a group of people that are different from each of the other patterns. Such clusters can then be evaluated through classical regression methods, interpreted and validated from a fluid intake point of view. Cluster analysis has been used extensively to evaluate dietary patterns [32], and is now being applied to fluid intake patterns in adults [17, 33, 34] and children [35–39], although its use is still limited.

However, to the best of our knowledge cluster analysis of fluid intake patterns in children and adolescents has only been performed in single-country population groups. A global analysis of FI patterns has not been possible to date due to variations in the methodology used to collect data; therefore, the importance of the country of residence per se has not been investigated using cluster analysis. With the availability of a validated 7-day fluid record [40] and a harmonized methodology across various countries [41–43] it is now possible to study global fluid intake patterns taking into consideration this variable. Therefore, the primary aim of this study was to identify different patterns of fluid intake in children and adolescents in six countries. The secondary aim was to characterize these patterns in terms of socio-demographics and lifestyle indicators.

## Materials and methods

### Survey and survey sample

This pooled secondary analysis was performed on the individual data of 3214 participants aged 4–17.9 years from six cross-sectional surveys. The primary objective of all surveys was to assess the intake of drinking water and different types of beverages. The surveys included in the pooled analysis were conducted in Latin America (Mexico, Brazil, Argentina, Uruguay), and Asia (China, Indonesia) in 2016. The recruitment of participants and further details of the populations included in this analysis have been described previously [41–43].

Participants and parents were selected from a database of individuals volunteering for population surveys or via a systematic door-to-door approach with an invitation for their child to participate. All participants, including parents and children, willing to join in the survey received detailed information about the survey objectives, what was expected from them, as well as a disclosure of the survey’s provisions to preserve confidentiality, risks and benefits, and a clear explanation about their option to participate voluntarily or not in the survey. After offering a detailed description of the survey, parents were asked for oral approval for their child to participate. No monetary incentive was offered for taking part in the survey. All data were recorded anonymously. Therefore, participants cannot be identified directly, or through identifiers linked to the participants. The survey protocol was reviewed and approved by the University of Arkansas Review Board (ref. 14-12-376).

### Fluid assessment

Participants were provided with the Liq.In^7^ record, a 7-day fluid-specific record validated for accuracy and reliability [40]. The Liq.In^7^ record was presented in the official country language. In all countries, except China, a paper version of the record was delivered and explained to the participants during an interview at home. After a period of 7 days, the record was collected by the researcher and checked for completion with the participant. In China, participants completed an electronic version of the Liq.In^7^ record via a smart phone. Participants were instructed to report all drinking events at any moment of the day with details such as the fluid type, size of the container from which the fluid was drunk and the actual volume consumed. Food consumption was not recorded. To assist participants in estimating the precise volume of fluid consumed, a photographic booklet of standard fluid containers supported the records. For children younger than 12 years, the primary caregiver was responsible for completing the record.

### Anthropometric and lifestyle variables

The anthropometric and lifestyle data were collected via a questionnaire during a face-to-face visit at home, except in China where these data were collected via a web-based questionnaire. In all countries, except for Indonesia, height (m) and weight (kg) were self-reported by participants or caregivers depending on the participant’s age. In Indonesia, weight was measured by the survey researchers. The body mass index (BMI) was calculated (kg/m^2^); BMI classification was based on sex- and age-specific cut-off values for individuals aged ≤ 18 years [44].

To determine the socioeconomic status (SES) of each participant, country-adapted classifications were applied. In Mexico, Argentina and Uruguay, the Asociación Mexicana de Agencias de Investigación de Mercado y Opinión Pública (AMAI) system was used and in Brazil the ABEP classification [45, 46]. Both systems use a combination of the following criteria to determine the SES of a household; work status, occupation, education, medical coverage, number of domestic servants, number of bathrooms, household equipment, possession of an international credit card and/or access to public utility services. In Indonesia, SES was determined based on the combined score of household expenditure, electricity usage, household cooking equipment and the kind of source of drinking water (e.g., tap water, well water, spring water) for use in the household. In China, SES was determined based on the household income of the participant. An income of RMB ≤ 5999 was classified as SES DE, an income between RMB 6000 and 11,999 as SES C and an income ≥ RMB 12,000 as level AB. For this analysis, SES classes were harmonized as detailed in Table S1 of the online resources.

The number of hours per day the participant spent watching TV or any content on a screen (≥ 2 or < 2 h/day) was considered as a proxy for sedentary behavior. The frequency of any exercise or sports, independent of the intensity, was recorded. To evaluate the availability of fluids at school, the following question was included in the questionnaire: “At school, is there a drinking fountain of water, a vending machine for beverages and/or a snack shop inside school available?” Moreover, the use of a lunchbox was assessed with the question “How often do you prepare a lunchbox for your child to go to school?” All variables and their modalities are listed in the Online Resource Table S2.

### Classification and analysis of fluid types

The fluids recorded were classified into the following eight categories: water (tap and bottled water); milk and milk derivatives; hot beverages (coffee, tea and other hot beverages); 100% fruit juices; SSB (carbonated soft drinks (CSD), juice-based drinks, functional beverages, e.g., energy and sports drinks, ready to drink tea and coffee and flavored water); artificial/non-nutritive sweetened beverages (A/NSB) (diet/zero/light soft drinks); alcoholic beverages and other beverages. Full details are given in Online Resource Table S3. Volumes of all categories were summed to give TFI.

### Statistical analysis

**Fig. 1:**
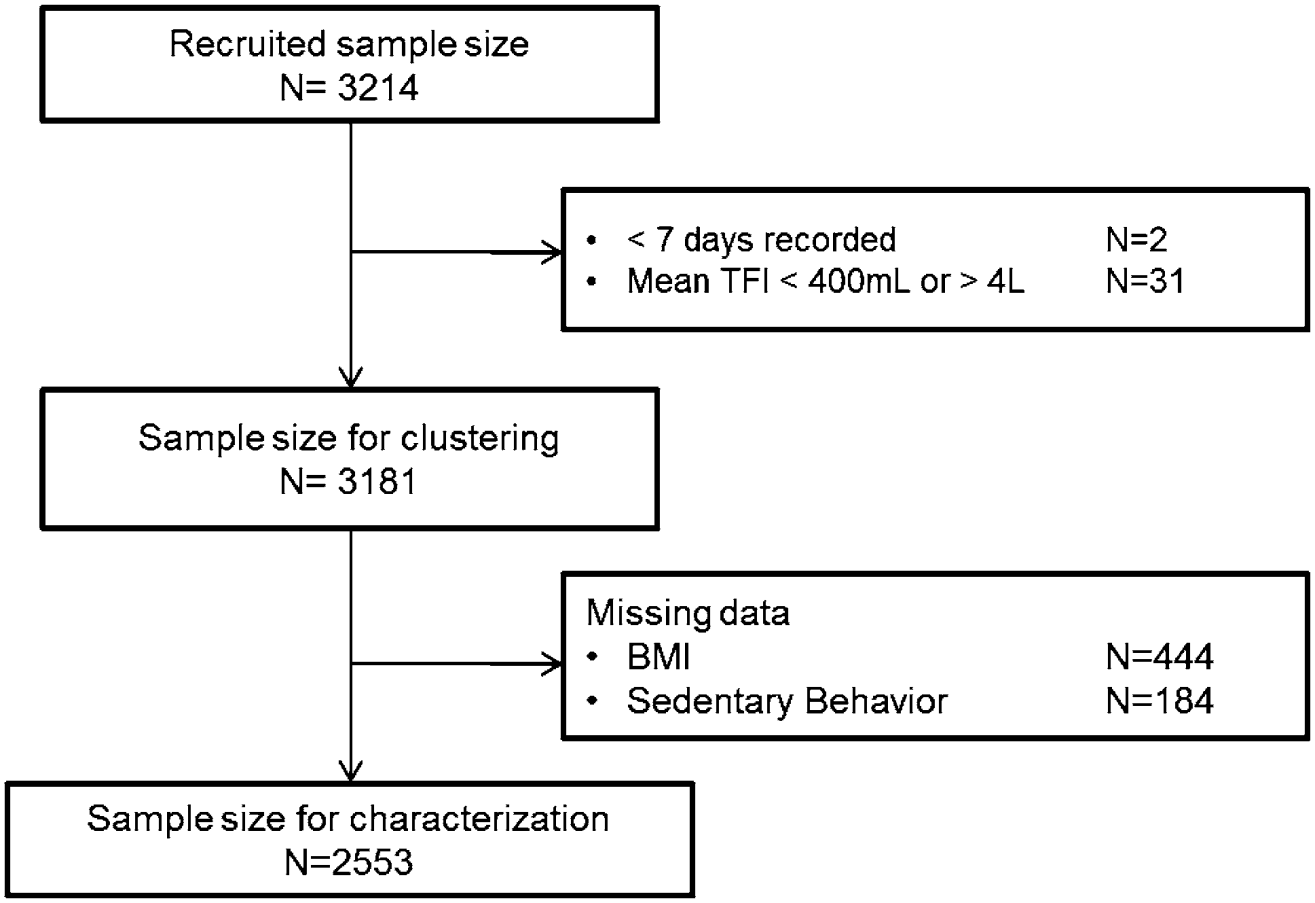
Flowchart showing sample selection for clustering and characterization

Participants who did not complete the full 7-day fluid record, and/or participants reporting a mean total daily fluid intake below 0.4 L/day, physiologically the minimum daily urine production [47], or higher than 6 L/day, were excluded from the analysis (Fig. 1). Only participants who had all socio-demographic and lifestyle indicators were included in the characterization of the cluster. Consequently, 628 subjects were excluded from the characterization (Fig. 1). The partitioning around medoids (PAM) method of R package ‘Cluster’ [48] was applied to the mean intake of the eight fluid types. PAM method is an unsupervised clustering method which needs an a priori number of clusters. To select the optimal number of clusters, a two-step approach was applied: the first step was to test several clusterings with different number of clusters from 2 to 50. For each clustering, the average silhouette coefficient, a statistical indicator to evaluate the robustness of the clustering, was computed (Online resources Fig S1). Only those clusterings with an average silhouette coefficient greater than 0.25 and a sample size balanced enough between clusters were considered statistically robust enough to go on to the second step, being the interpretability check. Remaining clusterings were then evaluated from a fluid intake point of view and the most interpretable one was chosen as the optimal clustering. Interpretability was evaluated by the predominant fluid intake within a cluster and the difference between clusters.

For fluid intake comparisons between clusters, a one-way ANOVA was used with clusters as factors. To correct for multiple testing over the different fluid types, Benjamini–Hochberg correction was used. When significant, Tukey post hoc tests were used to determine which clusters were different. ANOVA and post hoc tests were performed using JMP^®^ v12 software (SAS Institute Inc., Cary, NC, USA). For characterization of clusters with socio-demographics and lifestyles indicators, the “catdes” function of FactoMineR [49], an R package, was used. For stringency purposes, clusters’ characteristics were selected as being significant at a 0.1% level and ranked by their *v* test from the highest to the lowest. The *v* tests can be interpreted in the same way as *Z* scores: the higher the *v* test, the stronger the characteristic. Positive *v* test means an over-representation and negative an under-representation. Chi-square tests were used to evaluate differences between clusters for categorical variables (Online Resource Table S2).

## Results

### Identification of drinking patterns

**Fig. 2:**
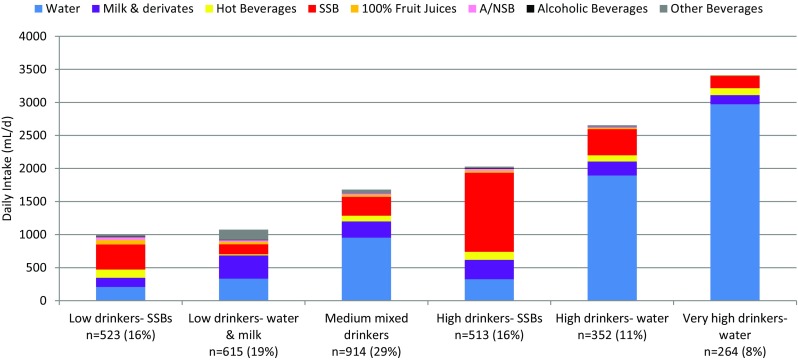
Mean daily intake of different fluid types (mL/day) of each cluster among children and adolescents. *SSB* sugar-sweetened beverages, *A*/*NSB* artificial/non-nutritive-sweetened beverages

Fifteen clusters showed a silhouette coefficient greater than 0.25 (Online resources Figure S1). Different numbers of clusters were explored before selecting an analysis based on six fluid patterns (clusters), as this was the most interpretable based on fluid intake amount/composition. The daily total fluid intake (TFI) of each cluster is shown in Table 1 and Fig. 2. Predominant fluid volume and fluid type for each cluster are highlighted in bold in Table 1. The six fluid patterns were: *low drinkers–SSB* (*n* 523; 16%), *low drinkers–water and milk* (*n* 615; 19%), *medium mixed drinkers* (*n* 914; 29%), *high drinkers–SSB* (*n* 513; 16%), *high drinkers–water* (*n* 352; 11%) and *very high drinkers–water* (*n* 264; 8%). The largest cluster was medium mixed drinkers, which represented 29% of the total sample.

**Table 1:** Mean (± SD) daily intake of different fluid types (mL/day) within clusters in children and adolescents from 4 to 17 years

Fluid type	Low drinkers–SSB (*n* = 523)	Low drinkers–water and milk (*n* = 615)	Medium mixed drinkers (*n* = 914)	High drinkers–SSB (*n* = 513)	High drinkers–water (*n* = 352)	Very high drinkers–water (*n* = 264)
Water	209 ± 152^e^	**332** ± **166**^**d**^	**952** ± **267**^**c**^	325 ± 281^d^	**1893** ± **265**^b^	**2972** ± **635**^a^
Milk derivatives	139 ± 126^d^	**353** ± **264**^a^	247 ± 270^c^	294 ± 283^b^	212 ± 291^c^	139 ± 207^d^
Hot beverages	123 ± 230^a^	16 ± 46^c^	88 ± 194^b^	121 ± 208^a^	95 ± 172^ab^	103 ± 211^ab^
SSB	**381** ± **173**^b^	152 ± 144^d^	285 ± 273^c^	**1197** ± **512**^a^	394 ± 435^b^	184 ± 273^d^
100% Fruit juices	66 ± 206^a^	39 ± 77^b^	29 ± 88^bc^	27 ± 81^bcd^	17 ± 55^cd^	7 ± 34^d^
A/NSB	44 ± 157^a^	25 ± 129^abc^	22 ± 101^bc^	35 ± 134^ab^	12 ± 56^bc^	1 ± 12^c^
Alcoholic beverages	8 ± 46^ab^	3 ± 48^ab^	3 ± 38^ab^	10 ± 59^a^	0 ± 5^b^	1 ± 9^b^
Other beverages	31 ± 78^c^	155 ± 212^a^	53 ± 141^b^	21 ± 82^c^	30 ± 106^bc^	4 ± 33^c^
Total fluid intake^a^	1000 ± 431	1076 ± 420	1681 ± 552	2028 ± 739	2654 ± 642	3411 ± 771

Numbers in bold indicate the predominant fluid type in each cluster

One-way ANOVA was performed with Tukey HSD post hoc test to evaluate the differences between clusters. Clusters not identified by same letter are significantly different (*p* < 0.05)

*SSB* sugar-sweetened beverages, *A*/*NSB* artificial/non-nutritive-sweetened beverages

^a^Illustrative variable in clustering

### Comparisons between drinking pattern clusters

Mean water intake was significantly different between clusters (Table 1). The *low drinkers–water and milk* cluster had a significantly lower water intake compared with the *medium mixed drinkers, high drinkers–water* and the *very high drinkers–water*, and a higher water intake compared with the cluster *low drinkers–SSB*. No significant difference in water intake was observed between the *low drinkers–water and milk* and *high drinkers–SSB clusters*. The *low drinkers–water and milk* cluster consumed significantly more milk than any of the other clusters and had a significantly lower intake of hot beverages compared with all the other clusters. No significant difference in hot beverage intake was observed between the *high drinkers–SSB, low drinkers–SSB, high drinkers–water* and *very high drinkers–water* clusters and between the *medium mixed drinkers, high* and *very high drinkers–water* clusters. The *high drinkers–SSB* cluster had a significantly higher intake of SSB compared with all the other clusters. *Low drinkers–SSB* and *high drinkers–water* clusters had significantly higher SSB intakes compared with the *medium mixed drinkers*, the *low drinkers–water and milk* and *the very high drinkers–water*.

### Characterization of the fluid intake patterns

The characteristics of the six FI patterns are shown in Table 2(a–f). Country of residence was the dominant characteristic in all six patterns. The *low drinkers–SSB* cluster (Table 2(a)) had an over-representation of Brazilians (33%), Mexicans (27%) and to a lesser extent Argentinians (22%). Indonesians were under-represented in this cluster (2%).

**Table 2:** Characteristics ranked according to *v* test of the children and adolescents present in the clusters

Variable	Modality	Cluster/modality (%)	Ranking *v* test
*Low drinkers*–*SSB* [*n* 523 (17% of total sample)] (a)			
Country	Brazil	33	7.8
Country	Mexico	27	7.0
Country	Argentina	22	3.4
Country	Indonesia	2	− 14.2
*Low drinkers–water and milk* [*n* 615 (19% of total sample)] (b)			
Country	China	51	18.7
Sedentary behavior	< 2 h/day	26	9.5
Lunchbox frequency	Daily or nearly	30	7.4
Age group	4–9 years	26	7.4
Access to fluid at school	Yes	23	6.0
Lunchbox frequency	2–5/week	28	4.7
Socio-economic status	AB	25	3.3
Country	Argentina	9	− 5.3
Lunchbox frequency	Never or rarely	13	− 6.3
Age group	10–17 years	14	− 7.4
Sedentary behavior	≥ 2 h/day	12	− 9.5
Country	Indonesia	3	− 14.9
*Medium mixed drinkers* [*n* 914 (29% of total sample)] (c)			
Country	Indonesia	38	5.6
Country	Argentina	22	− 3.5
Country	Mexico	20	− 5.3
*High drinkers*–*SSB* [*n* 513 (16% of total sample)] (d)			
Country	Argentina	44	13.3
Country	Uruguay	32	6.9
Sedentary behavior	≥ 2 h/day	20	6.3
Socio economic status	DE	21	5.6
Access to fluid at school	No	24	5.0
Lunchbox frequency	Never or rarely	20	4.3
Country	Mexico	22	4.0
BMI classification	Overweight	20	3.9
Socio-economic status	AB	9	− 4.8
Frequency physical activity	2/month to 1/week	9	− 5.0
Access to fluid at school	Yes	12	− 5.6
Lunchbox frequency	Daily or nearly daily	9	− 6.1
Sedentary behavior	< 2 h/day	11	− 6.3
Country	China	4	− 9.3
Country	Indonesia	1	− 15.1
*High drinkers*–*water* [*n* 552 (11% of total sample)] (e)			
Country	Indonesia	29	17.1
Sedentary behavior	≥ 2 h/day	14	4.1
Frequency physical activity	2/month to 1/week	16	3.6
Sedentary behavior	≤ 2 h/day	9	− 4.1
Country	Brazil	4	− 4.6
Country	Uruguay	3	− 5.4
Country	Argentina	3	− 6.0
Country	China	3	− 7.4
*Very high drinkers*–*water* [*n* 264 (8% of total sample)] (f)			
Country	Indonesia	27	20.8
Socio-economic status	C	11	4.5
BMI classification	Underweight	20	4.4
Frequency physical activity	2/month to 1/week	12	3.4
Sedentary behavior	≥ 2 h/day	10	3.4
Sedentary behavior	< 2 h/day	7	− 3.4
Lunchbox frequency	2 to 5/week	4	− 3.7
Frequency physical activity	2/week to 1/day	6	− 3.9
Socio-economic status	DE	5	− 4
Access to fluid at school	No	3	− 5.1
Country	Uruguay	1	− 5.7
Country	Brazil	1	− 5.7
Country	Mexico	2	− 6.3
Country	Argentina	1	− 6.6
Country	China	0	− 9.5

The most significant characteristic associated with *low drinkers–water and milk* (Table 2(b)) was again the country of residence, with 51% of Chinese participants represented in this cluster. Other characteristics over-represented in this cluster included having sedentary behavior approximated by a screen time of more than 2 h/day, being of higher SES class (AB), frequently having a lunch box (daily or nearly daily), being in the younger age group (4–9 years) and having a fluid source available at school. The characteristics that were under-represented in this cluster were residing in Argentina (9%) or Indonesia (3%). Adolescents (ages 10–17 years) and the children who did not have a lunchbox prepared daily by their parents were also under-represented in this cluster.

The country of residence was the only significant characteristic of the *medium mixed drinkers* cluster (Table 2(c)). Indonesian participants were over-represented in this cluster, with 38%. In this cluster, Argentinian and Mexican were both under-represented.

Residents in Argentina, Uruguay and Mexico were over-represented in the *high drinkers–SSB* cluster (Table 2(d)) with 44, 32 and 22% of these countries’ participants, respectively. The other characteristics over-represented in this cluster were children and adolescents from the lowest socioeconomic class (DE), having a sedentary behavior ≥ 2 h/day, never or rarely taking a lunchbox to school nor having available fluids at school and being overweight. Chinese and Indonesian residents were significantly under-represented in this cluster with only 4% from China and 1% from Indonesia.

The defining characteristics of the *high drinkers–water* cluster (Table 2(e)) were country and physical activity. Twenty-nine percent of Indonesians were in this cluster compared with 4% from Brazil and 3% each from China, Uruguay and Argentina. Participants who reported being physically active once a week to twice monthly were over-represented in this cluster as was sedentary behavior of 2 or more hours/day.

The *very high drinkers–water* FI pattern cluster (Table 2(f)) had an over-representation of Indonesians, the other five countries being under-represented. The other characteristics that were over-represented in this cluster were being in SES C, being underweight and having a physical activity level of once a week to twice a month.

## Discussion

Cluster analysis enables the identification of behaviors and associated characteristics, which may help target the specific populations that require intervention to change behavior. Unlike other cluster analyses, that have looked at fluid intake types in the context of energy content [35, 38], or diet quality [36], the emphasis of this analysis was drinking behavior. A total of eight fluid types were used in this cluster analysis to identify fluid intake patterns in children and adolescents across six countries. The first observation was that the FI patterns in this analysis were driven by SSB and water, and to a lesser extent milk and its derivatives. The second most striking observation was that the most significant characteristic across the FI patterns was country of residence. The Latin American countries were more represented in the *low drinkers–SSB* or the *high drinkers–SSB* FI patterns. These results are consistent with reports based on volumes of fluid types consumed [21, 42]. High intakes of SSB in Latin American countries have already raised concerns given the associations with dental caries [50], obesity and overweight [51] and associated metabolic conditions among children and adolescent [52]. In addition to high SSB intake, residence in Brazil, Mexico and Argentina was associated with low total fluid intake (*low drinkers–SSB*). The mean total fluid intake for this cluster was 1 L/day, which compares unfavorably with recommendations on the adequate intake of water from fluids [53]. Children and adolescents in these countries may be at risk of suboptimal hydration [54], which is associated with impaired cognition and low mood [55] and physical performance [56]. Uruguayan children and adolescents had high TFI and were over-represented in the *high drinkers–SSB* pattern. Therefore, interventions may be most effective if targeted at replacing energy-containing drink with water while maintaining the TFI. This analysis suggests that interventions in these countries should be targeted at increasing drinking water consumption while reducing sugar fluid intake. Some countries, including Mexico [57], are adopting this approach by introducing taxes to increase the price of sugar drinks; it is possible that this will particularly influence those in lower SELs [58].

Chinese residents were predominantly in the *low drinkers–water and milk* FI cluster (51%); a pattern in which the younger children (4–9 years) were over-represented; as were taking a lunch box to school and having fluid available at school. This pattern appears to reflect the policies implemented in China whereby a school lunch that includes a serving of 250 mL of milk is provided to first and middle-school children [59, 60]. Indonesians were over-represented in both the *high drinkers–* and *very high drinkers–water* patterns; these FI patterns have TFIs > 2.5 L/day as shown in a previous analysis [41]. From the present analysis, it would appear that in terms of fluid volume and type, there is little of concern that requires intervention in these latter two clusters. This could be due to all proactive actions undertaken in Indonesia to increase the access to safe water [61] and to encourage water consumption [62]. However, with increasing levels of obesity [63] and type 2 diabetes [64] in Indonesia there are concerns about increasing SSB consumption [65]. Public health policies and interventions are needed to halt and hopefully reverse this trend [66].

Fluid pattern analyses in children and adolescents have been conducted in the USA [30] and Canada [35]. Bougatsas et al. identified six clusters [30] including one which was similar to the *low drinkers–water and milk* cluster. Other comparisons between the two analyses are difficult due to the differences in fluid type classification. The analysis by Danyliw and colleagues [35] identified five clusters including milk and high-fat milk; however, water was excluded from the analysis as fluids were categorized on the basis of their energy and nutrient content. Other analyses have concentrated entirely on energy content rather than volume of fluid types. This is the first study to include data from more than one country; therefore, direct comparisons to other analyses are difficult and reasons for any similarities or differences are beyond the remit of the present analysis. However, while societal and cultural influences on food patterns are well recognized [67], identification of the factors that influence drinking patterns requires further research.

Socioeconomic level was a significant characteristic in three of the FI clusters. The *low drinkers–milk and water* cluster was more represented by SES AB, *high drinkers–SSB* were associated with SES DE and *very high drinkers–water* were associated with SES C. Disparities in SES and hydration [68], water [69] and type of fluids consumed [70] have been reported although evidence from cluster analysis is limited and complicated by the lack of consistent definitions of SEL. Danyliw et al. [35] noted differences in intake according to household security and income, but no specific patterns were established. In a recent systematic review lower SES was associated with higher SSB consumption [71]. In the present analysis, a lower SES was also a significant characteristic of the *high intake–SSB* cluster. Therefore, it would seem appropriate for interventions in those countries that aim to decrease SSB consumption and replace SSB with water, target children and adolescents in the lower SELs.

Sedentary behavior and/or physical activity were significant characteristics of four of the FI pattern clusters. Participants who reported less than 2 h of sedentary behavior were over-represented in the *low drinkers–water and milk* cluster. Children and adolescents who reported more than 2 h of sedentary behavior were over-represented in the *high drinkers with SSB* cluster; those who reported being physically active once a week to twice a month were under-represented. A recent cluster analysis of data from the ELANA and HELENA studies in children and adolescents reported clusters characterized by sedentary behavior and SSB consumption [72]. This is not surprising given the established links between sedentary behavior and less healthy dietary intake including SSB consumption [71, 73, 74]. Those who reported sedentary behavior greater than 2 h a week and/or being physically active once a week to twice a month were over-represented in the *very high drinkers–water cluster*. While this may appear contradictory, this phenomenon has been reported before and is probably due to those who report being sedentary for two or more hours per day compensating by being physically active at other times of the day [72]. Clearly, the prevention and treatment of overweight and obesity in children and adolescents requires a multifaceted approach, which focuses on changing dietary habits, including reducing SSB consumption, and reducing sedentary behavior and or increasing physical activity.

The current study has several strengths including the use of a harmonized sampling and data collection methodology across the countries and of a validated assessment method for total fluid intake [40], reflecting the day-to-day behavior of the participants over a 7-day period. The sample size and the use of data from participants in six countries undoubtedly strengthened the analysis. Objective statistical criteria such as using the silhouette coefficient to identify the number of clusters combined with the subsequent use of subjective criteria rendered the selected clusters interpretable. However, it is important to recognize the limitations of this study. Missing data for some of the variables resulted in a slightly reduced sample size; however, this is inevitable in large cross-sectional studies such as the Liq.In^7^ survey. The use of biomarkers for hydration status or health outcome measures would have strengthened the findings and possible implications of the analysis. Parents or primary carers recorded fluid intake and responded to questions about lifestyle and socio-demographics for younger participants, while this may have increased precision they may have been biased towards demonstrated healthy characteristics. In addition, adolescents were not asked if their parents provided a lunchbox for school nor about water availability in schools. While the questions on sedentary behavior and physical activity provide vital and interesting information, it would have been better to have used a validated physical activity questionnaire such as the International Physical Activity Questionnaire (IPAQ). To ensure a reliable and sensitive approximation of socioeconomic status, country-specific methods were used as a harmonized classification system is not currently available.

This analysis is the first to investigate fluid intake patterns across countries and has shown that country of residence is an important determinant of cluster membership. Therefore, it would be interesting to repeat the analysis within each country and extend the survey to other countries and regions of the world. Given the interest in establishing guidance and recommendations across regions, e.g., Latin American countries, or continents, e.g., Europe, once more data are available it would be interesting to repeat the analysis again within these regions. Cluster analysis of fluid intake patterns could be a useful tool for monitoring interventions aimed at increasing water intake while reducing SSB consumption by repeating the analysis over a period of time.

## Conclusions

This analysis identified six clusters with differing fluid intake patterns, which varied in terms of total fluid intake. The consumption of water and SSB was the primary drivers of the clusters. Country of residence proved to be an important variable, with some countries being over- or under-represented in the clusters. In addition, socio-demographic and lifestyle factors played a role in determining the characteristics of each cluster. Together this information highlights the need to target interventions in particular populations aimed at changing fluid intake behavior and improving health, e.g., increasing water intake and reducing SSB consumption in Mexico. This analysis emphasizes the need for more local surveys to provide valuable data for the development, implementation and evaluation of policies and programs aimed at changing fluid intake behavior.

## Electronic supplementary material

Below is the link to the electronic supplementary material.

Supplementary material 1 (DOCX 56 KB)
