# Supplementary material for: Fluid intake patterns of children and adolescents: results of six Liq.In7 national cross-sectional surveys
Source: Eur J Nutr. 2018 Jun 1;57(Suppl 3):113–23. doi: 10.1007/s00394-018-1725-y (PMC6008348; doi:10.1007/s00394-018-1725-y)
Supplement: Supplementary file 1 — Supplementary material 1 (DOCX 56 KB) [file 394_2018_1725_MOESM1_ESM.docx]

Fluid intake patterns of children and adolescents: results of six Liq.In^7^ national cross-sectional surveys.

*Morin C, ^1^ Gandy J, ^2,3^ Brazeilles R, ^4^ Moreno LA,^5,6^ Kavouras SA,^7,8^ Martinez H,^9,10^ Salas-Salvadó J,^6,11^ Bottin J,^1^ Guelinckx I,^1*^*

^1^ Department of Hydration & Health, Danone Nutricia Research, Palaiseau, France

^2^ British Dietetic Association, Birmingham, UK

^3^ School of Life Medical services, University of Hertfordshire, Hatfield, UK

^4^ Biometrics & Data Science Department, Danone Research, Palaiseau, France

^5^ GENUD (Growth, Exercise, Nutrition and Development) Research Group, Faculty of Health Sciences, Universidad de Zaragoza, Instituto Agroalimentario de Aragón (IA2), Instituto Investigación Sanitaria Aragón (IIS Aragón)

^6^ CIBERobn (Centro de Investigación Biomédica en Red Fisiopatología de la Obesidad y Nutrición), Institute of Health Carlos III, Madrid, Spain

^7^ Hydration Science Lab, University of Arkansas, Fayetteville, AR, USA

^8^ Division of Endocrinology, University of Arkansas for Medical Sciences, Little Rock, AR, USA

^9^ Nutrition International, Ottawa, Canada

^10^ Hospital Infantil de México Federico Gómez, México City, México

^11^ Human Nutrition Unit, Hospital Universitari de Sant Joan de Reus, Faculty of Medicine and Health Sciences, Institut d’Investigació Sanitària Pere Virgili, Biochemistry and Biotechnology Department, Universitat Rovira i Virgili Reus, Spain

**Corresponding author:** Isabelle GUELINCKX, Hydration and Health department, Danone Research, Route Départemental 128, 91767 Palaiseau, France; isabelle.guelinckx@danone.com

**ONLINE RESOURCES**

**Table S1** Harmonization of the classification of socioeconomic status

| Country | Socioeconomic status | Socioeconomic status for analysis |
| --- | --- | --- |
| Mexico | AB | AB |
|  | C+ | C |
|  | C | C |
|  | C- | C |
|  | D+ | DE |
|  | D | DE |
|  |  |  |
| Sao Paulo* | A (45 - 100 points) | AB |
|  | B1 (38 - 44 points ) | AB |
|  | B2 (29 - 37 points ) | AB |
|  | C1(23 - 28 points ) | C |
|  | C2 (17 t- 22 points ) | C |
|  |  |  |
| Argentina | AB | AB |
|  | C1 | C |
|  | C2 | C |
|  | C3 | C |
|  | D1 | DE |
|  | D2 | DE |
|  |  |  |
| Uruguay | ABC1 | AB |
|  | C2 | C |
|  | C3 | C |
|  | D1 | DE |
|  |  |  |
| China | Grade C (HHI:RMB12000 or above) | AB |
|  | Grade B (HHI: RMB6000-RMB11999) | AB |
|  | Grade A (HHI:RMB5999 or below) | C |
|  |  |  |
| Indonesia | High | AB |
|  | Medium | C |
|  | Low | DE |

*Socioeconomic status DE was excluded for recruitment in Sao Paulo (Brazil)

**Table S2** List of the socio-demographics and lifestyle variables used for the characterization of each cluster

| **Variables** | **Modality** |
| --- | --- |
| Country | Mexico |
|  | Brazil |
|  | Argentina |
|  | Uruguay |
|  | China |
|  | Indonesia |
|  |  |
| Age group | 4-9 years |
|  | 10-17 years |
|  |  |
| Gender | Boy |
|  | Girl |
|  |  |
| BMI classification | Underweight |
|  | Normal |
|  | Overweight |
|  | Obese |
|  | No data |
|  |  |
| Frequency of physical activity | 2/week - 1/day |
|  | 2/month - 1/week |
|  | 1/month or less often |
|  | Not recorded |
|  |  |
| Sedentary behavior | ≥ 2h/day |
|  | < 2h/day |
|  |  |
| Socioeconomic status | AB |
|  | C |
|  | DE |
|  |  |
| Access to fluid at school* | Yes |
|  | No |
|  |  |
| Lunchbox frequency* | Daily or nearly |
|  | 2 to 5/week |
|  | Once a month to once a week |
|  | Never or rarely |

***** *Only in children ≤12 yr*

**Table S3** Classification of the fluid types

| **Classification of fluids** | **Detailed Fluid types** |
| --- | --- |
| **Water** |  |
| *Bottled water* | Unflavored still water, unflavored sparkling water, still water jug/gallon/carafe |
| *Tap water* | Tap water, filtered water, boiled water |
|  |  |
| **Milk & derivatives** | Low fat and full fat milk, raw milk, regular/powder/syrup flavored milk (ready-to-drink or homemade), yogurt milk, atole/champurrado, powder milk, fruit shake with milk, cocoa compound with milk, vegetal milk (soya, almond…), sweetened condensed milk, milk tea (e.g. Assamu), |
| **Hot beverages** |  |
| *Coffee* | Coffee, coffee from coffee maker, powder instant coffee, vending machine coffee, restaurant/franchise coffee |
| *Tea* | Homemade hot/cold tea (from tea bags), infusions (herbal), restaurant/franchise tea, traditional herbal tea |
| *Mate* | Mate, mate cocido |
| *Other hot beverages* | Hot Beverages other than coffee, tea or maté (e.g. Amargos (Uruguay)) |
| **SSB** |  |
| *CSD* | Cola regular, concentrated/powder juice with sparkling water, flavored water sparkling, fruit flavored sparkling |
| *Juice-based drinks* | Eskimo/smoothies (ready to drink or homemade), packaged fruit juice/nectar/vegetable, Fruit shake with water, powder/syrups/concentrated water, still lemonade (ready to drink or homemade), kids fruit drinks, nectar |
| *Functional beverages* | Sports drinks, tonic regular, energy drinks, flavored water enriched with vitamins/minerals, quina water, protein drinks, liquid/powder isotonic |
| *RTD Tea & Coffee* | Bottled coffee, ice coffee, ready to drink bottled tea, ready to drink/homemade ice tea, powder tea, bubble tea |
| *Flavored water* | Flavored packaged/homemade water, aguas frescas, coconut water, clear flavored water, infused/herbal/vegetable water |
|  |  |
| **100% fruit juices** | Natural juice (vegetable/fruit), bottled 100% fruit/vegetables juice, homemade freshly squeezed juice, ready to drink or "take from outside" freshly squeezed juice |
|  |  |
| **A/NSB** | Cola light/zero, flavored water zero/light, fruit flavored light, light bottled tonic, light bottled ice tea, bottled juice light |
|  |  |
| **Alcoholic beverages** | Beer, cocktails, pure whiskey/vodka/gin/rum, spirit/liquor/digestive, wine, champagne, cider, rice wine |
|  |  |
| **Other beverages** | Beverages identified by participants as “other than listed above”, packaged soy drinks, agua de arroz (Mexico), diet drinks as meal replacement (slim fast), ready to drink soy based juice, beer 0% alcohol, vinegar drink (e.g. Tiandi Yi Hao) |

SSB sugar sweetened beverages, CSD carbonated sweetened drinks, RTD ready to drink, A/NSB artificial/nonnutritive sweetened beverage

**Table S4 Anthropometric and lifestyle characteristics of the six clusters identified among children and adolescents**

| Variable | Modality | Low drinkers-  SSB | Low drinkers- water & milk | Medium mixed  drinkers | High drinkers- SSB | High drinkers- water | Very high drinkers- water |
| --- | --- | --- | --- | --- | --- | --- | --- |
| Country | Mexico | **118 (27%)** | 98 (22%) | *86 (20%)* | **97 (22%)** | 32 (7%) | *8 (2%)* |
|  | Brazil | **87 (34%)** | 43 (17%) | 71 (27%) | 47 (18%) | *10 (4%)* | *2 (1%)* |
|  | Argentina | **71 (22%)** | *30 (9%)* | *70 (22%)* | **142 (44%)** | *9 (3%)* | *2 (1%)* |
|  | Uruguay | 40 (15%) | 36 (14%) | 92 (36%) | **82 (32%)** | 7 (3%) | 2 (1%) |
|  | China | 61 (12%) | **265 (51%)** | 157 (30%) | *20 (4%)* | *17 (3%)* | *1 (0%)* |
|  | Indonesia | *13 (2%)* | *25 (3%)* | **283 (38%)** | *9 (1%)* | **219 (29%)** | **201 (27%)** |
| Age group | 4-9 year | 146 (13%) | **289 (26%)** | 328 (30%) | 151 (14%) | 109 (10%) | 84 (8%) |
|  | 10-17 year | 244 (17%) | *208 (14%)* | 431 (30%) | 246 (17%) | 185 (13%) | 132 (9%) |
| Gender | Boy | 193 (14%) | 249 (18%) | 403 (29%) | 233 (17%) | 165 (12%) | 135 (10%) |
|  | Girl | 197 (17%) | 248 (21%) | 356 (30%) | 164 (14%) | 129 (11%) | 81 (7%) |
| BMI classification | Underweight | 16 (12%) | 16 (12%) | 41 (31%) | 16 (12%) | 16 (12%) | **27 (20%)** |
|  | Normal | 208 (16%) | 246 (19%) | 393 (30%) | 183 (14%) | 168 (13%) | 116 (9%) |
|  | Overweight | 121 (15%) | 155 (20%) | 226 (29%) | **157 (20%)** | 81 (10%) | 51 (6%) |
|  | Obese | 45 (14%) | 80 (25%) | 99 (31%) | 41 (13%) | 29 (9%) | 22 (7%) |
| Frequency of physical activity | 2/week to 1/day | 138 (15%) | 201 (23%) | 262 (29%) | 150 (17%) | 90 (10%) | *50 (6%)* |
|  | 2/month to 1/week | 54 (11%) | 102 (20%) | 156 (31%) | *43 (9%)* | **81 (16%)** | **62 (12%)** |
|  | 1/month or less often | 2 (4%) | 11 (22%) | 16 (32%) | 3 (6%) | 8 (16%) | 10 (20%) |
|  | Not recorded | 196 (18%) | 183 (16%) | 325 (29%) | 201 (18%) | 115 (10%) | 94 (8%) |
| Sedentary behavior | ≥ 2h/day | 171 (15%) | *141 (12%)* | 351 (29%) | **245 (20%)** | **172 (14%)** | **126 (10%)** |
|  | < 2h/day | 219 (16%) | **356 (26%)** | 408 (30%) | *152 (11%)* | *122 (9%)* | *90 (7%)* |
| Socioeconomic status | AB | 76 (15%) | **127 (25%)** | 166 (32%) | *46 (9%)* | 60 (11%) | *37 (7%)* |
|  | C | 175 (14%) | 219 (18%) | 353 (29%) | 173 (14%) | 156 (12%) | **134 (11%)** |
|  | DE | 139 (17%) | *151 (18%)* | 240 (29%) | **178 (21%)** | 78 (9%) | 45 (5%) |
| Access to fluid at school | Yes | 225 (14%) | **362 (23%)** | 475 (30%) | *192 (12%)* | 177 (11%) | 131 (8%) |
|  | No | 73 (16%) | 88 (19%) | 144 (31%) | **109 (23%)** | 35 (7%) | *14 (3%)* |
|  | Not recorded | 92 (17%) | 47 (9%) | 140 (26%) | 96 (18%) | 82 (15%) | 71 (13%) |
| Lunchbox frequency | Daily or nearly | 108 (16%) | 197 (30%) | 186 (28%) | *57 (9%)* | 67 (10%) | 51 (8%) |
|  | 2 to 5/week | 70 (16%) | **119 (28%)** | 123 (29%) | 62 (15%) | 34 (9%) | *18 (4%)* |
|  | Once a month to once a week | 12 (12%) | 30 (29%) | 28 (27%) | 16 (15%) | 14 (13%) | 4 (4%) |
|  | Never or rarely | 108 (13%) | *104 (13%)* | 282 (34%) | **166 (20%)** | 97 (12%) | 72 (9%) |
|  | Not recorded | 92 (17%) | 47 (9%) | 140 (26%) | 96 (18%) | 82 (16%) | 71 (13%) |

Numbers in **bold** are significantly overrepresented in the cluster
Number in *italic* are significantly underrepresented in the cluster

**Figure S1** Statistical selection of the number of clusters based on the silhouette coefficient,
with a coefficient of 0.25 being the cut-off of statistical validity
